# Feasibility and Effectiveness of a Motion Tracking-Based Online Fitness Program for Office Workers

**DOI:** 10.3390/healthcare9050584

**Published:** 2021-05-14

**Authors:** Sun-Young Joo, Chang-Bae Lee, Na-Young Joo, Chung-Reen Kim

**Affiliations:** Department of Physical Medicine and Rehabilitation, Ulsan University Hospital, University of Ulsan College of Medicine, Ulsan 44033, Korea; 0734982@uuh.ulsan.kr (S.-Y.J.); 0734211@uuh.ulsan.kr (C.-B.L.); 9200615@uuh.ulsan.kr (N.-Y.J.)

**Keywords:** COVID-19, exercise, fitness, machine learning, physical activity

## Abstract

The development of technology-based home fitness has emerged from the booming digital healthcare market and recent demands for at-home fitness and health equipment due to the COVID-19 pandemic. Digital healthcare company Alyce Healthcare recently developed Weelo, which is a web-based online fitness program. Weelo recommends an exercise protocol through machine-learning-enabled recognition of the user’s motion and provides visual and auditory feedback. We evaluated whether Weelo improves physical and mental well-being to assess its capabilities and effectiveness. Thirty-two participants performed a total of 20 exercise sessions following the Weelo guide on a laptop. The participants were evaluated using a before and after exercise program, body composition, handgrip strength, six-minute walk test, modified star excursion balance test, short form 36, fatigue severity scale, Beck depression index, and a satisfaction survey. Overall, there was a significant improvement in muscle strength, endurance, and balance ability, as well as an improved quality of life and significant reduction in fatigue and depression. Participants showed high motivation to continue following the Weelo exercise program. In conclusion, utilizing Weelo improved physical and mental well-being and is considered to be an individual-use indoor exercise program that serves as an alternative to traditional face-to-face exercise.

## 1. Introduction

Interest in online fitness has rapidly increased due to advancements in technology and the expansion of the digital health care market [1,2]. Technology-based interventions confer several advantages over traditional face-to-face approaches, as they are often cost-effective, enable continuous self-monitoring, and are easily accessible, thus reducing the barriers of transportation and time [3,4]. The unprecedented lockdown due to the COVID-19 pandemic has increased consumer and developer awareness of the need for online home fitness, not only to overcome restrictions to at-home spaces and the lack of accessibility to exercise programs equipment but to solve the physiological and psychological health problems that arise when there are limitations to physical activity [5].

The exergame has been well-studied as an alternative to traditional exercise [3,6,7,8,9]. An exergame is a skill-driven physical activity that requires the participant to exercise in order to win the game [10]. Representatively, there are Wii™ and Xbox^®^ Kinect exergames. Recently, at-home fitness company Mirror began providing a paid online home fitness service using the smart mirror. In addition, there are mobile phone applications that guide proper exercise and provide self-monitoring. Interestingly, these exergames and mobile phone-based interventions produce similar effects as traditional exercise programs [11,12]. Peng et al. reported that exergaming significantly increased heart rate, VO_2_, and energy expenditure with similar effects as traditional physical activities [12].

However, technology-based exercises have several limitations. First, Wii™ games, Xbox^®^ Kinect games, and smart mirrors carry a significant cost burden for equipment and software purchases. Exergame equipment that is not completely portable inevitably has spatial and time constraints. Although smartphone applications are easily accessible, more convenient, and cost-effective, the small screen sizes of smartphones hinder interactive exercise with the application. To compensate for these shortcomings, startup company Alyce Healthcare developed a web-based exercise program, Weelo, which is accessible via a laptop or personal computer (PC). Weelo provides an appropriate personal exercise program that allows users to exercise interactively in real time and enables self-monitoring. Conveniently, users can use their own laptop or PC with a monitor that is much larger than their smartphone screen, so they can enjoy exercising anytime, anywhere, at a lower cost.

Therefore, Weelo seems to be a good alternative to traditional face-to-face exercise programs and the previously available exergames. For this study, the authors aimed to evaluate Weelo’s capability and effectiveness in improving physical and psychological well-being.

## 2. Materials and Methods

### 2.1. Subjects

From September to December 2020, office workers between the ages of 20 and 49 were enrolled in this prospective pre-post interventional study. The sample size was estimated based on the “General Health” domain of the Short Form-36 Health Survey questionnaire (SF-36). According to the “Short Form-36 Health Survey manual & interpretation guide,” a minimum of 27 participants were needed when the significance level (α) was 0.05, the power was 80%, and the number of points difference was 10 [13]. The recruitment posters were placed in our hospital and in nearby companies, and all subjects participated voluntarily. Subjects who had difficulty participating in exercise due to musculoskeletal or neurologic disorders or visual and auditory abnormalities were excluded. In addition, those who take drugs for mental disorders, such as depression or insomnia, were excluded. The Ulsan University Hospital institutional review board approved this study protocol (UUH 2020-01-005). This trial was registered with the Clinical Research Information Service (KCT0005023).

### 2.2. Weelo

**Figure 1:**
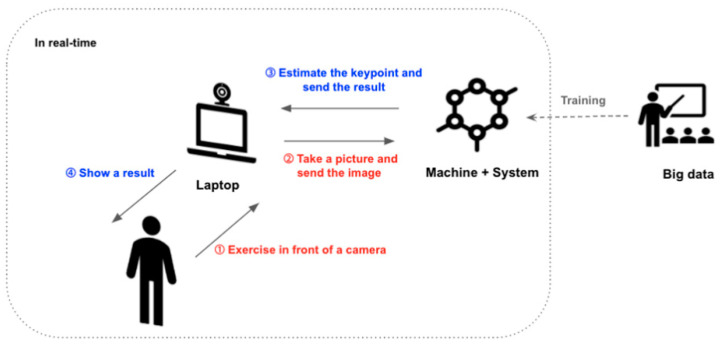
Scheme of the Weelo exercise program.

Weelo (Alyce Healthcare, Seoul, Korea) is a motion-tracking-based online fitness program that provides appropriate exercise programs and methods. Users can monitor their activity level and performance through real-time feedback. Weelo runs on a laptop or a PC. Although users can install the program to their device, users can also access the program through the Weelo website. Body movements are recognized using a camera device, but a regular webcam or built-in laptop camera are sufficient; therefore, no additional purchases for peripheral devices are necessary (Figure 1).

**Figure 2:**
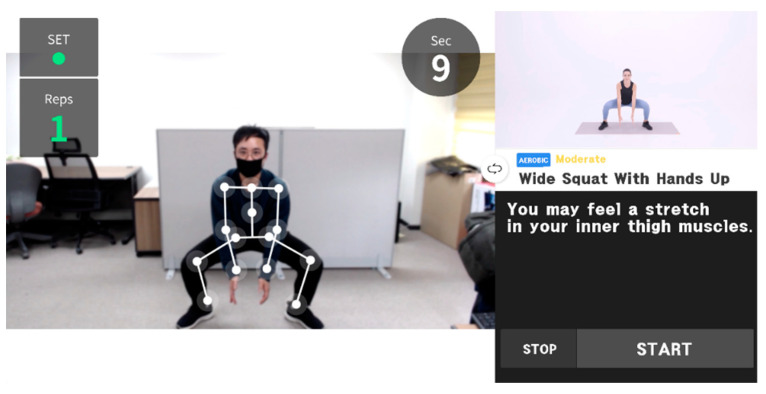
User interface of Weelo. The user’s real-time image and exercise-related data are displayed on the left, and exercise guide images and explanations are displayed on the right.

The Weelo user interface consists of a real-time image of the user, a guide avatar of a fitness trainer, and a space for textual descriptions (Figure 2). Weelo displays the user’s image on the screen and analyzes motions that are recognized by the camera, then counts the number of motions and evaluates the accuracy of the movements based on the exergame tasks, then provides visual and auditory feedback on the user’s performance and activity level. Recognition of the user’s motion is learned through a continuous tracking system based on feature point extraction and part affinity fields using machine learning technology with top-down segmentation. A total of 17 body parts are recognized in real time. Depending on the user’s computer graphic processing ability, body movements can be tracked at 15 to 60 frames per second. The mean average precision (mAP) was verified against the validation subset val2017 dataset provided by Microsoft common objects in context datasets, which resulted in 0.683 mAP [14]. The Korean beta version is available through the Weelo website (https://weelo.fit (14 May 2021)).

### 2.3. Intervention

Exercise intervention was performed five times per week, at a total of 20 times in four weeks. A 15-inch laptop, yoga mat, and two dumbbells (1 kg dumbbells for women, 3 kg dumbbells for men) were provided. Dumbbell weights were determined based on our own pilot study. To minimize the involvement of fitness trainers and to reduce the risk of injury from strong workouts, aerobic and strengthening exercises of light-to-medium level were provided. We prepared 14 types of aerobic exercise, 19 types of strength exercise, and 15 types of stretching exercise (Table 1). Every day, three exercises were randomly selected in each exercise category. To avoid the negative effects of the redundancy (i.e., boredom) of the exercise program, we changed the two exercises for each exercise category. However, the training level provided during the entire exercise program was set to be approximately the same among participants. Visual and auditory explanations were also provided within 1 min prior to the start of each exercise, and there was a 10-s break between sets.

The daily exercise time provided was about 30 to 45 min. Aerobic exercise was performed for about 20 min, and each exercise was repeated in two sets for 6 to 7 min. Strength training was performed in two sets of 15 repetitions for each exercise, and it took about 10 min. Dumbbells were used in some strengthening exercises. Stretching exercises were performed for 5 to 10 min; two sets of each exercise were performed for 1 min, and sometimes participants had to complete stretching exercises alternately on both sides.

The reference value of motion for each exercise was obtained by two fitness trainers and one physiotherapist. Visual and auditory feedback of bad, good, and excellent movement accuracy were provided during exercise. Compared to the reference value, the difference was approximately 8% or more for bad, 6% to 8% for good, and less than 6% for excellent. The angles that were measured were different depending on the motion. For example, in performing squats, the angles of the shoulder, hip, knee, and ankle were measured.

### 2.4. Assessments

Participants were evaluated before and four weeks after participation in the exercise program. Clinical data were collected, and the level of physical activity over the past week was investigated using an international physical activity questionnaire short form (IPAQ-SF) [15,16]. The pre- and post-assessment included data on the physical and functional status, quality of life (QOL), fatigue, and depression. A satisfaction survey was administered to the participants after completion of the exercise program.

### 2.5. Physical and Functional Status

Weight, body mass index (BMI), muscle mass, and body fat percentage were measured using an InBody 250 body composition analyzer (InBody, Seoul, Korea). Physical strength was evaluated based on the hand grip strength (HGS). The maximum HGS was measured by a handheld dynamometer (Saehan Corporation, MSD Europe Bvba, Belgium) with the elbow bent 90 degrees from the sitting position. The HGS task was repeated three times, alternating between right and left. The 6-min walk test (6MWT) is a simple tool for assessing exercise endurance and cardiopulmonary fitness [17]. The total distance traveled for 6 min on a 30 m course was measured. Dynamic balance was evaluated using a modified star excursion balance test (mSEBT) [18]. The subject stands on one foot and stretches the other foot so that the tip of the toe slightly touches the ground; then, the longest distance is measured. Each leg alternately performed the task in three directions: anterior, posteromedial, and posterolateral. To rule out the effect of difference in leg length, the % leg length (the measured distance ÷ the leg length ×100) was calculated, and distances in the three directions were averaged.

### 2.6. QOL, Fatigue, and Depression

The Korean version of SF-36 is a health-related QOL measurement tool consisting of 36 questions and eight scales (physical functioning, role—physical, bodily pain, general health, vitality, social functioning, role—emotional, and mental health) [13,19]. To evaluate the severity of fatigue, the Korean version of the fatigue severity scale (FSS) was used [20]. FSS is a self-report questionnaire that describes the fatigue levels from the previous week. FSS consists of nine items on a Likert-type scale of 1 to 7 points [21]. The Korean version of the Beck Depression Inventory-II (BDI) was used to evaluate the degree of depression [22,23].

### 2.7. Satisfaction Survey

A purpose-designed participant satisfaction survey consisting of 10 questions related to effectiveness, convenience, and level of satisfaction was completed at the end of the four-week exercise program. Of the 10 responses, eight were recorded on a five-point Likert scale. However, the perceived intensity of the exercise program and the type of exercise that was most satisfactory were selected in a three-choice method.

### 2.8. Statistical Analysis

Statistical Package for the Social Sciences (SPSS) version 24.0 was used for statistical analysis. The Kolmogorov–Smirnov test was used to test the assumption of normality, and all variables were distributed normally. The paired samples *t*-test was applied to determine the change in variables between pre- and post-participation in Weelo. Interpretations were made for data with a *p* value of ≤0.05, which was accepted as statistically significant.

## 3. Results

Out of the 35 participants, 32 completed the program. One participant dropped out due to sudden shoulder pain prior to beginning the program. Another participant was unable to continue due to trauma unrelated to this study. Another subject was excluded due to insufficient participation. According to the baseline IPAQ-SF, seven out of 32 participants actively participated in physical activity, and their mean energy expenditure was 788.56 ± 742.55 METs/week. However, in the case of remaining participants who were not physically active, the mean energy expenditure was 72.02 ± 120.01 METs/week. The general characteristics are presented in Table 2.

**Figure 3:**
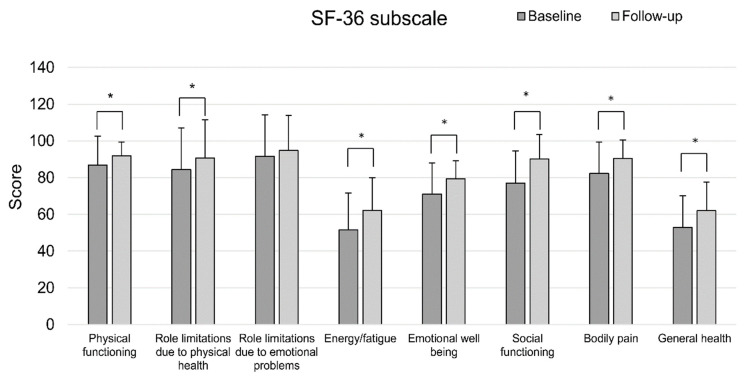
Comparison of the SF-36 scores in each domain between baseline and follow-up evaluation. * *p* < 0.05 is statistically significant.

Although there was no significant change in body composition after four weeks of exercise, HGS, 6MWT, and mSEBT showed significant improvement in physical performance (Table 3). In addition, the results of SF-36 showed significant improvement in QOL in all areas except the ‘role of limitations due to emotional problems’ (Table 3, Figure 3). On the FSS, participants complained of daily mild fatigue at baseline (3.86 ± 1.58), but after participating in the intervention, there was a significant improvement in fatigue (3.13 ± 1.14, *p* = 0.002). Based on the BDI, minimal depression was observed at baseline (7.00 ± 5.71), but significantly improved after intervention (4.06 ± 3.98, *p* < 0.001).

Based on the satisfaction survey (Table 4), participants thought that the exercise program was effective (81.25%) and the software program was easy to use (87.50%). In addition, 56.25% of the participants said the Weelo exercise program was better than other exercise programs they had experienced before. Although the auditory and visual explanation and feedback provided during exercise did not score highly, they were described as helpful, with the visual component seeming to be more helpful (53.12% vs. 43.75%). Very high agreement was observed for motivation to continue exercising (96.88%) after the program was completed. Even after the end of the exercise program, 50% of the participants actively wanted to continue following the Weelo program. Participants were most satisfied with the strengthening exercise and reported that the intensity of the exercise was adequate.

## 4. Discussion

The results of this study demonstrated that Weelo is a physically and psychologically effective home fitness program. Participants were guided to the recommended level of exercise during the study, and overall improvement of muscle strength, endurance, and balance ability was observed. Weelo improved the QOL, fatigue, and depression and provided users with high motivation to continue exercising.

To promote physical and psychological well-being, an exercise guideline for adults was recommended [24]. It is necessary to perform aerobic exercise of moderate intensity for at least 150 min per week or of vigorous intensity for at least 75 min per week, and to perform strength exercise at least twice per week. If the recommended exercise was performed by the study participants during the course of this study, physical function improved and mortality risk was reduced [25,26,27]. According to the suggested guideline, the authors tried to design an exercise type and duration to meet the classification of moderate intensity. After four weeks of active participation, muscle strength, endurance, and balance ability were significantly improved overall. Moreover, unlike the recent meta-analysis of the active video game, Weelo significantly increased the physical activity level, which seemed to mean that Weelo is more effective as an exercise than exergame [28].

In addition, QOL, depression, and fatigue symptoms also significantly improved. In fact, the positive effect of moderate-to-vigorous physical activity on QOL and psychologic well-being in elderly or cancer patients has already been established [29,30,31]. During the COVD-19 pandemic, the prevalence of depression symptoms increased more than threefold higher than before the emergence of COVID-19 in the United States of America [32]. In fact, the study period took place from November to December 2020, which was the middle phase of the pandemic crisis in South Korea. Although strict COVID-19 quarantine was not implemented in Ulsan, Korea during the study period and all participants continued their work as usual, there was some psychological stress due to restrictions on social and physical activities, such as restrictions on the use of multiple exercise facilities. Therefore, the baseline survey revealed that the participants complained of mild fatigue. However, the follow-up survey revealed an improvement in fatigue symptoms. Even though the baseline score for the BDI was minimal, significant improvements in depression symptoms were observed. Thus, Weelo seemed to be an appropriate exercise program for promoting physical and psychological well-being.

Above all, Weelo’s greatest strength is that it overcomes the spatial and time constraints of traditional exercise regimens. In general, exercise facilities have fixed locations and times of access, and often transportation is required to visit, which are inconveniences. Weelo is easily accessible from home or work at any time, regardless of the weather. In addition, as for other exergames, Weelo provides increased motivation for participating in the exercise. It is well-known that an important advantage of exergames is that they motivate sedentary people to exercise [7,33]. Likewise, in the post-study satisfaction survey, motivation to continue participating in the Weelo exercise program received the highest score.

In fact, the feasibility of similar exergames in combination with machine learning or virtual reality has been widely studied, and previous studies using exergames showed similar physical and psychological improvement [7,9,31,34]. Although the exergame study was mainly conducted on adolescent or elderly participants, direct comparison with Weelo was difficult. However, a study comparing elderly with home-based exercise showed that exergame was excellent in balance, functional mobility, and QOL [35]. However, there have been some limitations in clinical application. First, the requirement for consoles and accessories such as a smart mirror introduces a high cost burden. Making a purchase decision without knowing how often one will use the device, if at all, can be financially burdensome. In the case of Weelo, most users already have access to a laptop or PC at the home or the office; therefore, Weelo has a few extra costs besides the cost of the Weelo program. The only peripheral accessories required are a webcam and dumbbells, and the webcam is usually already included in the laptop or PC. Even if a webcam could be bought, it is much cheaper than most exergame accessories. Second, it was not easy to carry the consoles and accessories all the time, so they have spatial and time constraints. However, if an internet-connected laptop is available, Weelo users can participate in exercise anytime, anywhere, as long as there is a space of approximately 3 m × 3 m. Therefore, in situations of isolation, such as people under lockdown during the recent COVID-19 pandemic, Weelo may be a good alternative to conventional exergames as well as traditional gym fitness.

In addition, the results of the satisfaction survey showed that Weelo was satisfied with the exercise, was easy to operate, and showed high motivation for future participation in exercise. In particular, the high motivation for participation in exercise was similar to the reports of studies using smart mirror and exergames. However, it is not known whether such motivation and satisfaction can be sustained in the long term. Since previous studies using exergames suggested the risk of such a decrease in participation over time [36,37], efforts such as adjusting the difficulty of exercise and setting goals will be needed to induce continuous interest. Additionally, auditory and visual feedback were relatively low in satisfaction, so it seems that it is necessary to supplement to provide effective feedback in order to induce more interest.

Of course, Weelo has drawbacks. First, visual and auditory exercise descriptions and feedback were provided, but they are not superior to or similar to what a real physical trainer does. Reflecting this, the scores for auditory and visual feedback in the satisfaction survey were not higher than expected, because the participants were not familiar with this type of exercise guide. Due to technical limitations, it was difficult to provide detailed feedback for each participant. In particular, since posture was evaluated based on 2D images, it was still difficult to accurately identify the ideal posture in 3D reality. Therefore, customized exercise for each participant was not provided, and although the composition was changed randomly, almost the same exercise program was provided to participants. For this reason, even in the satisfaction survey about exercise intensity, 34.38% of participants felt that the intensity was inappropriate. Second, due to the location of the monitor, the exercise was inevitably composed of exercises consisting of motion facing the monitor. Due to technical problems, exercises performed by lying on the floor were not included. However, these shortcomings were expected to be overcome through technological improvement and acquired experience.

There were several limitations to this study. First the number of participants was small. Particularly, there might be differences based on covariates, such as sex, BMI, current physical activity participation, and exergaming experience. However, further analysis was difficult, owing to the small number of participants. In fact, the effects of these covariates are controversial. With respect to gender, women tended to think of exergame as an exercise, and reported that there were more positive psychological responses [38], but Soltani et al. reported that there are similar psychological effects between men and women [37]. Additionally, there were conflicting opinions on the influence of previous game experience [39,40]. Therefore, further studies on the effects of various confounding factors are required. Second, it was difficult to judge the superiority of conventional exercise, since there was no control group. However, since this was the first study on Weelo, we aimed to investigate the capability and effectiveness of Weelo rather than the superiority of a specific exercise method. However, in the future, we plan to conduct a study that will confirm the superiority of Weelo in comparison to traditional exercise or other exergames. Finally, since the long-term effects and sustainability of exercise were not evaluated, it was difficult to determine the long-term effects of Weelo. Finally, although the recent physical activity level was investigated with IPAQ-SF, the specific physical activity, sports or gaming experience that the subjects participated in was not investigated. However, since previous experiences might affect the immersion in this type of exercise, the authors should have checked the previous gaming and sports experience of participants. Particularly, previous researches on exergames still have controversy over the effect of game experience on energy expenditure [37,39,40], so further research is necessary.

In conclusion, this study evaluated the exercise usefulness of the Weelo online home fitness program and demonstrated the positive physical and mental effects. The participants who participated in an exercise program for four weeks showed improvements in muscle strength, endurance, balance ability, QOL, and symptoms of fatigue and depression. Participants reported that Weelo was an effective exercise program, and they also reported a high degree of motivation to continue participating in exercise. Therefore, Weelo is expected to provide an alternative exercise to traditional face-to-face exercise, which will help patients who are suffering from various diseases with limited outdoor activities and who are incapable of making personal contact with other people.

## Figures and Tables

**Table 1:** List of Weelo exercises.

Stretching Exercises	Strengthening Exercises	Aerobic Exercise
Quadriceps stretch Back stretch Side bend stretch Abdomen stretch Shoulder rotation stretch Overhead shoulder stretch Triceps stretch Cross shoulder stretch Chest stretch Rhomboids stretch Trapezius stretch Neck flexion stretch Neck extension stretch Neck rotation stretch Neck lateral flexion stretch	Isometric cervical flexion Isometric cervical extension Dumbbell bent-over row Y raise Shoulder external rotation Dumbbell shoulder press Arnold press Upright row Side lateral raise Front raise Bent over lateral raise Dumbbell curl Hammer curl Dead lift One leg dead lift Side band Squat Wide squat Side lunge Skate lunge	Jump twist Knee up Wide squat with hands up Toe touch Step jack with side lunge Step jack Side elbow Twist elbow Speed skaters Fly jack High knee Jumping jack Walking in place Running in place

**Table 2:** General characteristics of participants.

Characteristics	*N* = 32
Age (years)	35.26 ± 6.79
Male: Female	14:18
Height (cm)	168.59 ± 9.00
Right-handed: Left-handed	29:3
Married: Unmarried	20:12
Physically active: Non-active	7:25

Values are mean ± standard deviations.

**Table 3:** Comparison between pre- and post-intervention physical and psychological statuses.

Variables	Pre	Post	*p*
Body composition			
Body weight (kg)	69.65 ± 16.06	69.56 ± 15.97	0.697
Body mass index (kg/m^2^)	24.24 ± 3.76	24.23 ± 3.78	0.865
Skeletal muscle mass (kg)	27.69 ± 7.55	27.63 ± 7.42	0.431
Body fat (%)	28.33 ± 5.91	28.34 ± 6.01	0.951
Hand grip strength			
Right side (kg)	35.55 ± 13.21	39.61 ± 12.91	<0.001 *
Left side (kg)	33.76 ± 11.52	38.08 ± 11.83	<0.001 *
Modified star excursion balance test			
Right side (cm)	85.61 ± 10.04	94.33 ± 7.67	<0.001 *
Left side (cm)	85.81 ± 11.00	95.28 ± 8.29	<0.001 *
Aerobic capacity & endurance			
6-min walk test(m)	520.27 ± 48.9	559.02 ± 42.06	<0.001 *
Pain			
Visual analog scale	3.47 ± 2.77	2.78 ± 2.17	0.195
Short form 36			
Physical functioning	86.88 ± 15.70	91.88 ± 7.59	0.002 *
Role limitations due to physical health	84.38 ± 22.67	90.63 ± 20.82	0.041 *
Role limitations due to emotional problems	91.67 ± 22.40	94.79 ± 19.14	0.206
Energy/fatigue	20.63 ± 8.01	24.88 ± 7.11	<0.001 *
Emotional well-being	71.00 ± 16.94	79.38 ± 9.86	0.001 *
Social functioning	76.95 ± 17.71	90.23 ± 13.37	0.031 *
Bodily pain	82.27 ± 17.13	90.55 ± 9.95	0.004 *
General health	52.81 ± 17.22	62.03 ± 15.55	<0.001 *
Fatigue			
Fatigue severity scale	3.86 ± 1.58	3.13 ± 1.14	0.002 *
Depression			
Beck depression index	7.00 ± 5.71	4.06 ± 3.98	<0.001 *
International Physical Activity Questionnaire			
Vigorous (METs/week)	228.86 ± 456.56	577.54 ± 651.75	0.001 *
Moderate (METs/week)	276.27 ± 423.38	436.28 ± 300.61	0.022 *
Mild (METs/week)	796.16 ± 802.25	571.31 ± 361.37	0.089
Time spent sitting (min/day)	337.03 ± 219.63	337.13 ± 204.21	0.998

MET, metabolic equivalent task. Values are mean ± standard deviation. * *p* < 0.05 is statistically significant.

**Table 4:** Satisfaction survey results for Weelo.

I. Likert scale Questions	Strongly Agree	Agree	Neutral		Disagree	Strongly Disagree		Net Percent Agreeing (%)	Mean Score
Do you think that the Weelo exercise program was effective physically and mentally?	4	22	5		1	0		81.25	3.91
Do you think that the Weelo exercise program is better than the exercises that you have done before (home training, gym, yoga, etc.)?	3	15	10		5	0		56.25	3.5
Did the auditory explanation and feedback provided during the exercise program help you perform the exercise?	2	12	11		5	2		43.75	3.22
Did the visual explanation and feedback provided during the exercise program help you perform the exercise?	3	14	9		6	0		53.12	3.44
Was it easy to handle and operate the program with a laptop during exercise?	10	18	1		2	0		87.50	4.09
Did you feel that the exercise program was interesting?	3	12	11		5	1		46.88	3.34
If it is still available, would you like to use this program after your participation in the study is over?	6	10	13		2	1		50.00	3.56
After participating in the study, were you motivated to continue exercising?	11	20	1		0	0		96.88	4.31
II. Preference of exercise and intensity									
	Aerobic			Strengthening			Stretching		
What type of exercise program did you find most satisfying and helpful to you?	4 (12.5%)			21 (65.63%)			7 (21.88%)		
	Strong			Appropriate			Weak		
Was the exercise intensity of the provided exercise program appropriate for you?	7 (21.88%)			21 (65.63%)			4 (12.5%)		

## Data Availability

No new data were created or analyzed in this study. Data sharing is not applicable to this article.
